# Illumina MiSeq Sequencing Reveals Correlations among Fruit Ingredients, Environmental Factors, and AMF Communities in Three *Lycium Barbarum* Producing Regions of China

**DOI:** 10.1128/spectrum.02293-21

**Published:** 2022-03-02

**Authors:** Kaili Chen, Gang Huang, Yuekun Li, Xinrui Zhang, Yonghui Lei, Yang Li, Jie Xiong, Yanfei Sun

**Affiliations:** a College of Life Sciences/Xinjiang Production and Construction Corps Key Laboratory of Oasis Town and Mountain-basin System Ecology, Shihezi University, Shihezi, Xinjiang, China; b National Wolfberry Engineering Research Center, Wolfberry Science Research Institute, Ningxia Academy of Agriculture and Forestry Sciences, Yinchuan, China; c Department of Plant Protection, College of Agriculture, Shihezi University, Shihezi, Xinjiang, China; University of Massachusetts Amherst

**Keywords:** *Lycium barbarum*, arbuscular mycorrhizal fungi (AMF), soil physicochemical properties, enzyme activity, temperature, fruit ingredients

## Abstract

The symbiotic relationship of arbuscular mycorrhizal fungi (AMF) is important for *Lycium barbarum*, a highly nutritious and medicinal crop. However, the influence of environmental factors on AMF communities remains largely elusive. Based on MiSeq sequencing, we analyzed AMF communities in rhizosphere soils of *L. barbarum* with growth synchronization in three typical *L. barbarum* cultivation sites in China. The Zhongning region has poor soils with a high richness of AMF communities. Geographical environmental variances lead to differences in AMF communities which in turn affects the active ingredients of *L. barbarum* fruit. Furthermore, different genera of AMF showed significant correlations with environmental factors and fruit ingredients. The three genera, *Claroideoglomus*, *Dominikia,* and *Funneliformis* correlated to environmental factors and fruits ingredients in a similar manner affecting the whole sugar (TS) and flavonoids (FLA) contents in the fruits of *L. barbarum*. Also, these showed a significantly positive correlation with soil pH. This fact was unknown so far due to different soil acidity/alkalinity in different studies.

**IMPORTANCE** The climatic and ecological environment is a complex phenomenon, involving various environmental factors that regulate the diversity and population distribution structure of AMF communities affecting plant growth, crop composition, and yield. Current studies on the effects of environmental factors on AMF communities have mainly focused on soil conditions and host plants. Fewer studies have been conducted on the correlation between temperature, enzyme activity, plant fruiting, and AMF communities. The present study investigated the diversity of AMF communities and the influence of environmental factors on their distribution patterns, which showed similar effects on some AMF species. The results suggest that screening AMF fungicides that meet the target may significantly help soil restoration reducing the use of chemical fertilizers and a large amount of human and material resources.

## INTRODUCTION

*Lycium barbarum* L (wolfberry; Goji), originally cultivated in East Asia (1), has been used as a traditional medicinal herb and food supplement in China and other Asian countries for >2,000 years (2). Goji berries are known for hemopoietic, antiradiation, antiaging, anticancer, antioxidation, and immunity stimulant properties (3–5). Ningxia (especially the daodi region), with a semi-arid climate, is considered the best area to produce the best quality goji berries (6, 7). Studies have shown that different producing areas show great variation in the average yield of LBPs (*Lycium barbarum* polysaccharides) (8). This suggests that different cultivating regions can affect the functional content and fruit quality of goji fruits (9, 10). Also, the different Chinese growing areas face the problem of identical, complex, and variable pests due to reliance on foreign *L. barbarum* seedlings, and long-term cultivation induced damages. Notably, *L. barbarum* rhizosphere microorganisms play an important role in improving *L. barbarum* resistance to diseases.

Arbuscular mycorrhizal fungi (AMF, Glomeromycota) are the most widely distributed endophytic mycorrhizae, which constitute a group of root obligate biotrophs that exchange mutual benefits with most plants (11, 12). The community structure of AMF and their metabolites play an important role in improving nutrition, growth, immunity, and abiotic stresses resistance (13–18). AMF can expand the range of nutrient uptake by the host plant by altering its root morphology and mycelial network formation, releasing root secretions such as organic acids, phosphatases, and protons to alter soil structure and physicochemical properties, such as degrading insoluble phosphate in soil (19) and inducing specific expression of relevant phosphorus transporter protein genes (20) enhancing phosphorus uptake. Most importantly, studies showed that AMF is also associated with carbon sequestration (21) and increases soil organic carbon (SOC) deposition via secretion of glomalin-related soil protein (GRSP) and modulates plant carbon partition (22). The promotion of soil enzyme activity may be due to an increase in carbon and nutrient content in the plant roots. The difference in quantity and diversity at the genus level of the AMF communities leads to different amounts of root exudates. These differences lead to different soil invertase activity and cellulase activity (23). Besides, soil dehydrogenases are closely related to soil respiratory metabolism, citric acid cycle, and nitrogen metabolism and can be used to represent the metabolic activities of soil microorganisms. Also, soil urease activity is closely related to the nitrogen mineralization potential of the soil. In addition to soil physical and chemical properties and soil enzyme activities that are closely related to AMF communities, temperature changes can directly affect the AMF community composition. Given that different microbial taxa have different temperature ranges for growth, for example, higher temperature promotes microorganisms better suited to higher temperature environments, it also triggers physiological changes in microorganisms thereby affecting their carbon use efficiency. Temperature can also affect the structure and function of decomposer food webs by altering the quality of the apoplankton. It was shown that increases in temperature, nitrogen, and phosphorus deposition can alter AMF function by affecting AMF diversity and community composition (12). Also, the microbial communities in soils with large aggregates are more sensitive to soil warming (24). AMF significantly enhances the multifunctionality of the ecosystem. Therefore, studying how environmental factors affect the growth of AMF communities can provide new insights for sustained crop production (25).

Change in soil characteristics due to different locations, both in latitude and longitude directions (26, 27), can affect the ubiquity of indigenous AMF for plant ecosystems (28). It can significantly change AMF adaptation to local environments causing functional differences (12, 29). Also, the host plants can have a controlling effect on AMF colonization (30). So far, most studies have only focused on the sampling sites at different locations to examine the correlation between soil physicochemical factors and AMF communities (31–33). Apart from soil physicochemical properties, soil enzyme activity, soil temperature, soil moisture, and the relative contributions of relevant environmental factors such as altitude, relative air humidity, soil pH, and available phosphorus (P), kalium (K), and magnesium (Mg) can influence the AMF spore production and root colonization (32, 34–36). Also, different seasons have a significant impact on AMF diversity (37, 38). However, on sampling site locations and soil physicochemical properties, whereas the above-mentioned parameters have not given enough importance while studying the diversity of AMF communities in different habitats.

The traditional AMF taxonomic classification is mainly based on the morphological features of soil asexual spores, which has certain limitations (39, 40). Recent years have seen a growth in the number of AMF biodiversity studies performed by modern NGS-based methods, especially the Illumina MiSeq method (40). High-throughput sequencing has sped up the identification and relative quantification of the microbial community (41, 42).

**FIG 1:**
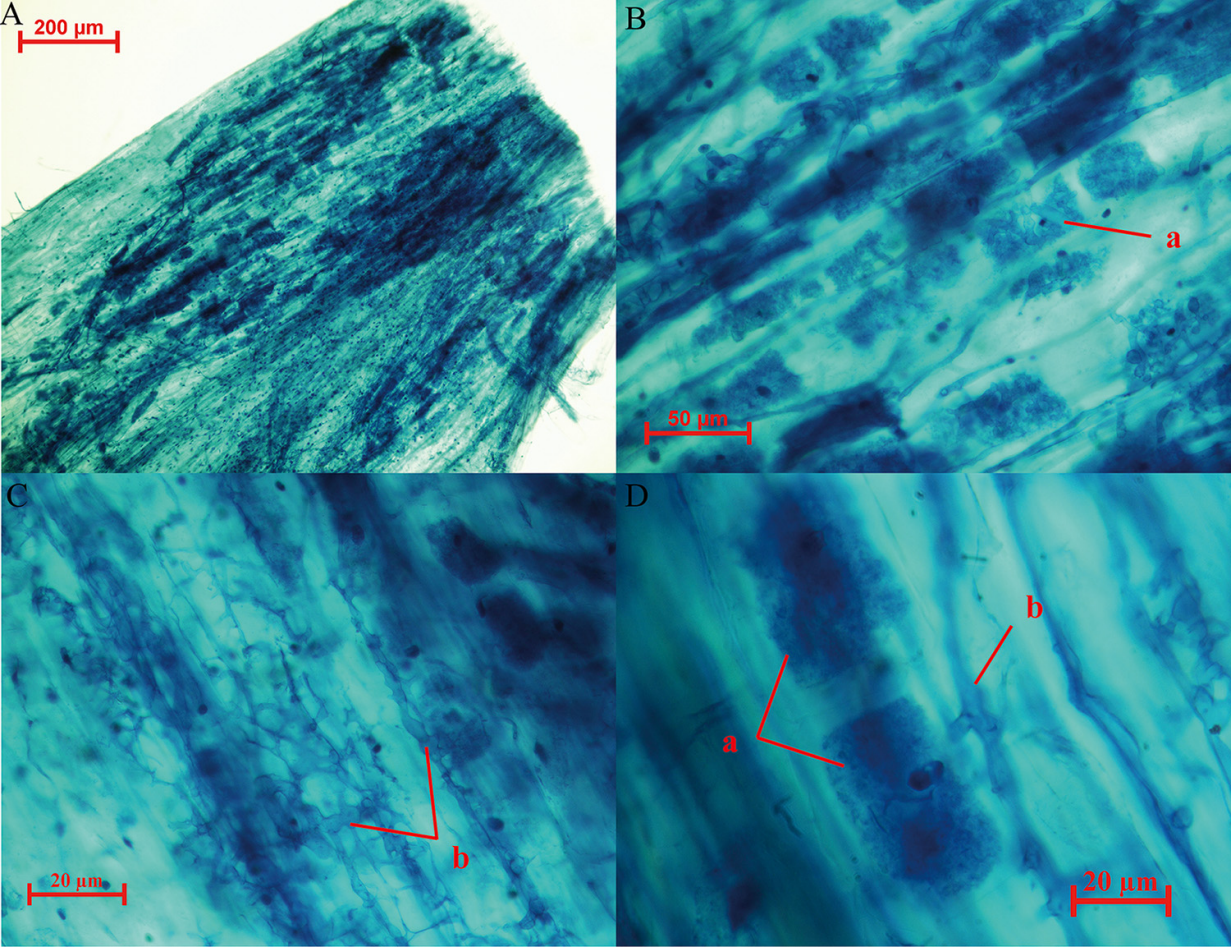
Trillium blue staining of root segments of *L. barbarum* sampled from three regions, A, B, C, D are different magnifications, respectively. (a) marks the typical arum-type structure produced by AMF infestation; (b) marks an AMF mycelium.

Before beginning this study, we collected root segments of *L. barbarum* from three areas and confirmed the symbiosis of mycorrhizal fungi (Fig. 1). In this study, we selected the characteristic and representative production areas of *L. barbarum* and then used respective rhizosphere soil samples for sequencing of ribosomal small subunit gene fragments to analyze the composition of AMF communities under different habitats. Also, we examined the impact of soil physicochemical properties, enzyme activity, climate temperature, and fruit ingredients. Understanding the change in AMF communities can provide a novel perspective for *L. barbarum* production under specific ecological conditions.

## RESULTS

### Comparison of environmental factors and *L. barbarum* fruit ingredients in three regions.

All the nine soil physicochemical indicators showed significant differences among the three regions in the various months, with all soils being alkalinesee (Table S1 in the supplemental material). From May to September, the soil pH rose, then fell, and finally ended up being slightly lower than the initial value. Notably, Zhongning (ZN) had the highest pH (alkaline) and the remaining eight indicators were lower than in the other two regions. In all three regions, total soil nitrogen (TN), total phosphorus (TP), and total potassium (TK) tended to decrease from May to September, while available nitrogen (AN) and available potassium (AK) increased and then decreased; the increase in available phosphorus (AP) was significantly higher in the ZN region. In May, cellulase (CEL) was significantly higher in the ZN region compared with other regions, while urease (URE) was the lowest. In contrast, sucrase (SUR) was significantly higher in the Dulan (DL) region than in the other two regions (see Table S2 in the supplemental material). Importantly, temperature data were less significantly distinct (see Table S3 in the supplemental material), with TS, *L. barbarum* polysaccharides (LBP), and FLA being significantly higher in the ZN region, betaine (BET) and FLA being highest in the DL region (see Table S4 in the supplemental material). *P* <0.05 was used to assess the significance of the data.

### rDNA sequencing and species classification.

The dilution curves for all samples from the three regions flattened out, and a further increase in sequencing depth only added a small number of additional species. This indicated that sequencing depth was adequate (see Fig. S1 in the supplemental material). The optimized sequence number of 1959297 was obtained from 27 soil samples and clustered into 1,762 microbial operational taxonomic units (OTUs), dominated by Ascomycota with 1126 OTUs, followed by 152 Basidiomycota, 66 Chytridiomycota, 55 Mortierellomycota, and 51 Glomeromycota OTUs, while other fungal phyla were relatively low. The AMF OTUs belonged to two classes, three orders, four families, nine genera, and 15 species. Fifty-one Glomeromycota OTUs belonged to five genera; *Rhizophagus*, *Dominikia*, and *Glomus* each had seven OTUs; *Funneliformis* and *Claroideoglomus* had three and two OTUs, respectively.

### AMF community richness and diversity.

**FIG 2:**
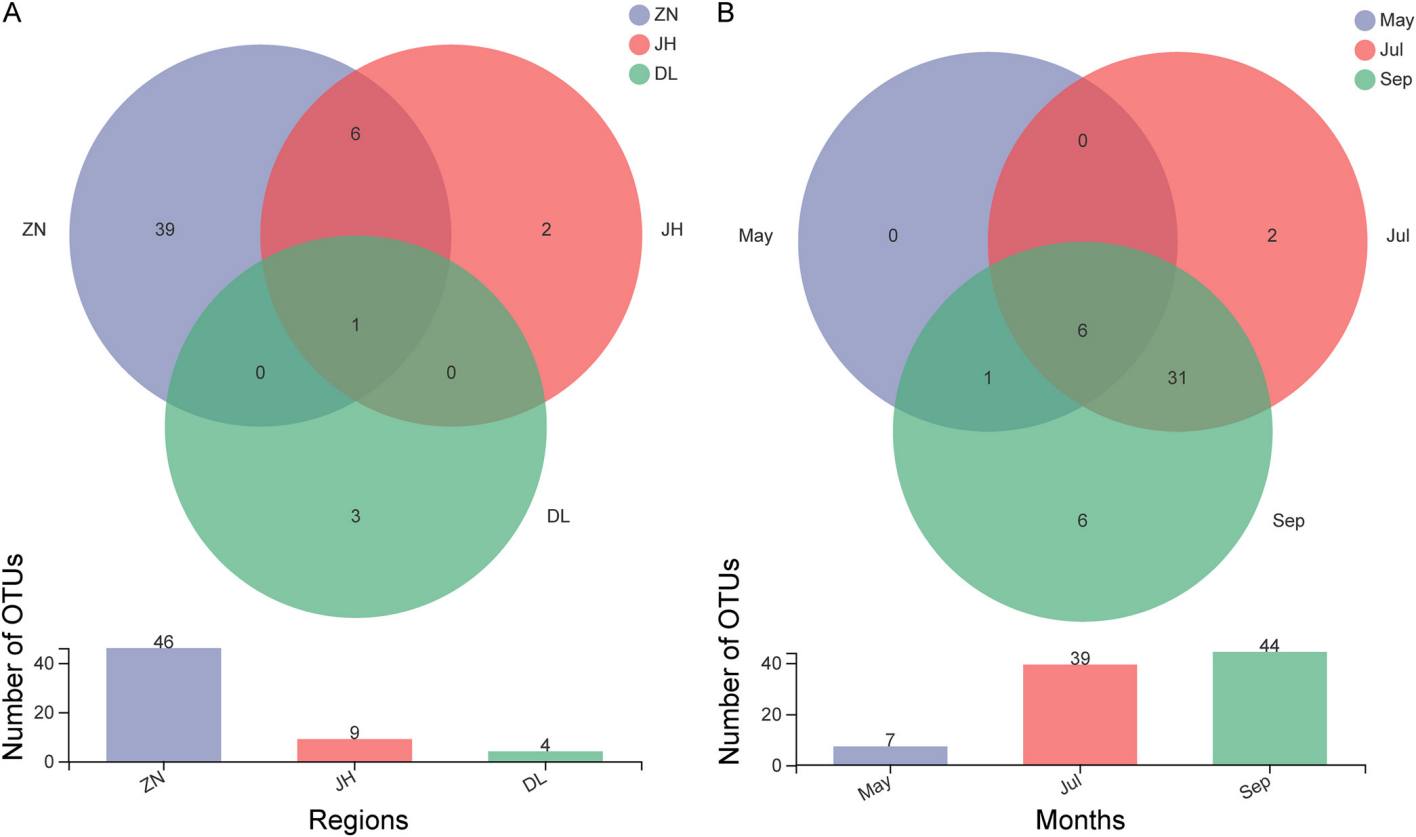
Venn diagrams of AMF based on operational taxonomic units (OTUs). (A) Represents the total OTUs differences in soil samples collected in the three regions for the three periods; (B) represents the OTUs differences for the 3 different month’s samples in the ZN region. The circles indicate the total number of OTUs in the respective sample, and the overlapping areas indicate the co-occurring OTUs in adjacent samples.

The community richness and diversity analyses revealed higher AMF abundance in ZN rhizosphere soil samples than in the Jinghe (JH) and DL samples (Fig. 2A). Among the ZN samples, AMF abundance was higher in July and September samples (Fig. 2B).

Community richness indices (S_obs_, ACE, and Chao) revealed significantly higher AMF community richness in the ZN region than in JH and DL; among ZN samples, richness was higher in July and highest in September (Table 1). The Shannon and Simpson indices for community diversity reflected better diversity in the ZN region, followed by the other two regions; among the ZN samples, the community diversity was higher in July and September than in May. Also, the ZN samples showed higher phylogenetic diversity (PD).

**TABLE 1:** Rhizosphere AMF diversity indices at a 97% identity threshold^*a*^

Sample	S_obs_	Shannon	Simpson	ACE	Chao	PD
ZN5	2.66 ± 0.22c	0.66 ± 0.33b	0.54 ± 0.24a	11.38 ± 8.01b	3.83 ± 1.74c	1.21 ± 0.20b
ZN7	25.66 ± 0.33b	3.05 ± 0.02a	0.04 ± 0.00a	31.67 ± 0.93a	29.25 ± 1.25b	2.77 ± 0.10a
ZN9	32.00 ± 1.73a	3.14 ± 0.05a	0.04 ± 0.00a	40.31 ± 1.92a	37.91 ± 1.50a	3.10 ± 0.25a
ZN	20.10 ± 4.49A	2.28 ± 0.42A	0.20 ± 0.11A	27.78 ± 4.91A	23.66 ± 5.17A	2.36 ± 0.31A
JH	1.66 ± 0.78B	0.47 ± 0.21B	0.08 ± 0.04A	5.30 ± 3.85B	2.61 ± 1.34B	0.52 ± 0.22B
DL	1.00 ± 0.29B	0.21 ± 0.11B	0.24 ± 0.11A	1.01 ± 0.45B	1.00 ± 0.29B	0.54 ± 0.14B

a Three regions Zhongning (ZN), Jinghe (JH), and Dulan (DL) showed significant differences in rhizosphere AMF diversity (indicated by upper case letters, *P* < 0.05). Among ZN samples, May, July, and September samples showed significant variations (indicated by lower case letters, *P* < 0.05). Values are mean ± standard error.

### AMF community structure and diversity.

**FIG 3:**
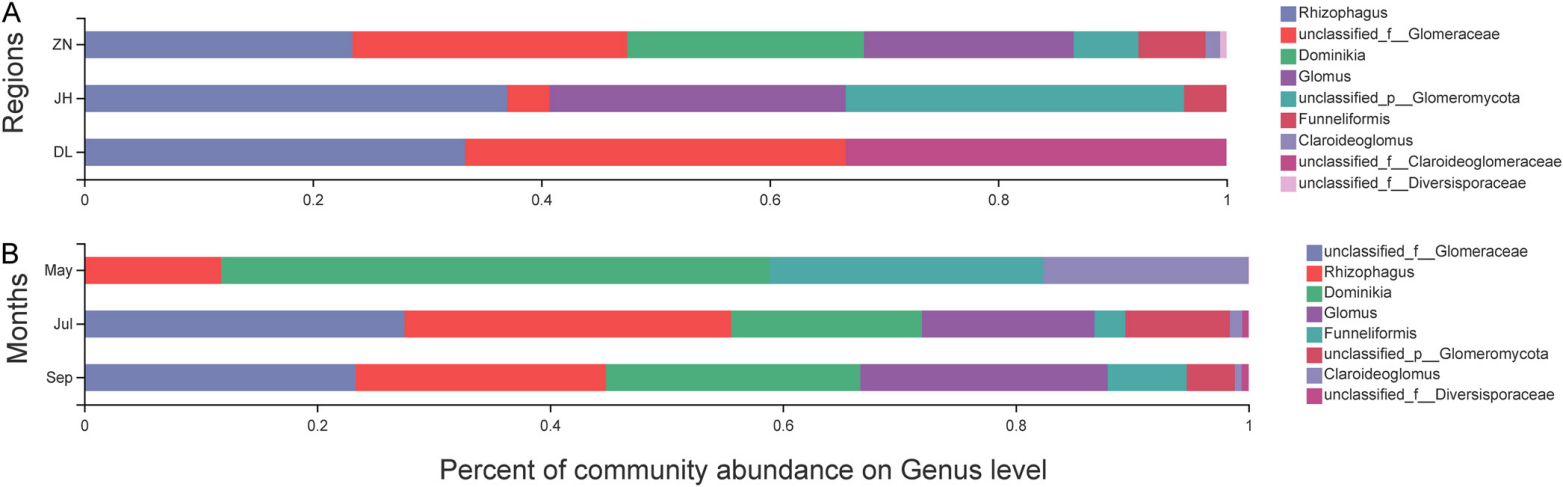
Soil AMF community distribution at the genus level. (A) Total soil samples from three regions; (B) ZN soil samples from 3 months. Species with abundance <0.01% were assigned to other groups.

**FIG 4:**
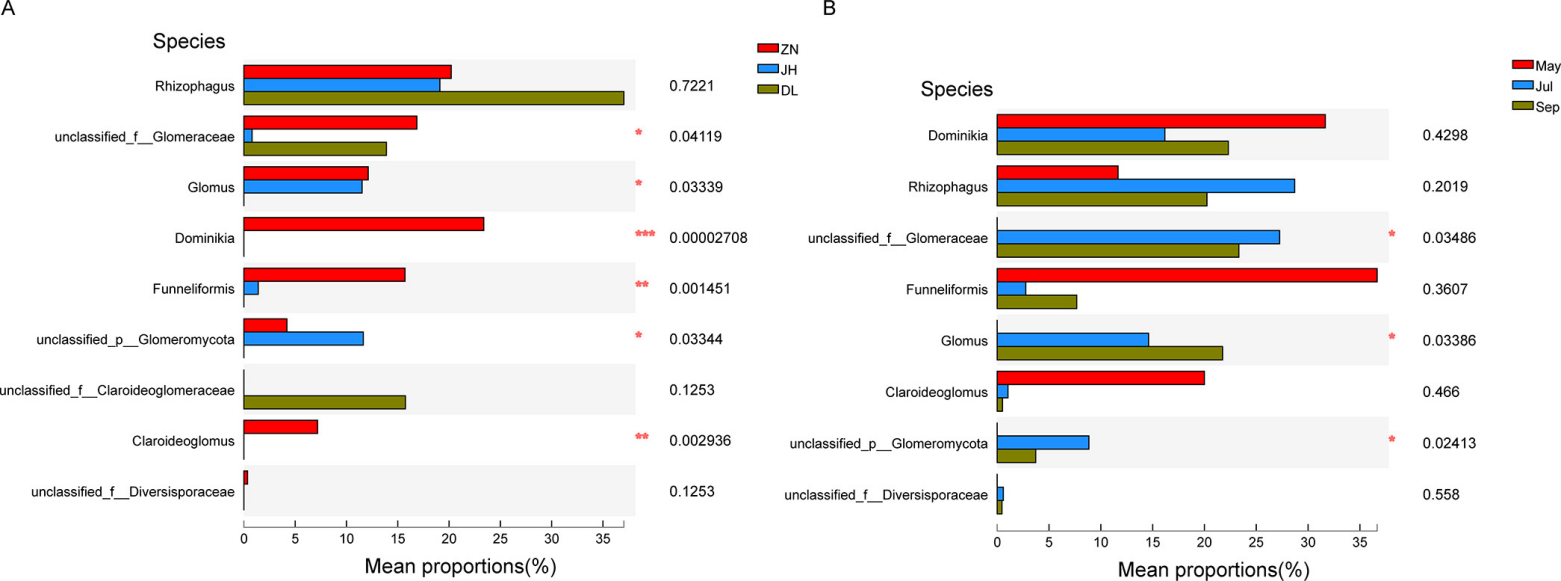
Kruskal-Wallis H test bar plot for samples from three regions and 3 months in the ZN region. (A) Genera enrichment in samples from three regions and (B) ZN samples in 3 different months. The *y* and *x* axes indicate species names at a given taxonomic level and mean relative abundance, respectively. Colored bars indicate different groupings, and the *P* values are mentioned on the right side. *, 0.01 < *P* ≤ 0.05; **, 0.001 < *P* ≤ 0.01; ***, *P* ≤ 0.001.

Soil samples from the three regions were analyzed for significant differences in the AMF community composition at the genus level. In total, five AMF genera were identified with classifiable sequences; the non-classifiable sequences were assigned to other families. Rhizophagus accounted for 23%, 37%, and 33% of the total sequences in ZN, JH, and DL samples, respectively (Fig. 3A). The genus *Dominikia* and *Claroideoglomus* were extremely enriched in ZN samples than the other two region samples (Fig. 4A). The July and September ZN samples showed a greater variety of genera (Fig. 3B), with significantly different species compared to the May ZN sample (Fig. 4B).

**FIG 5:**
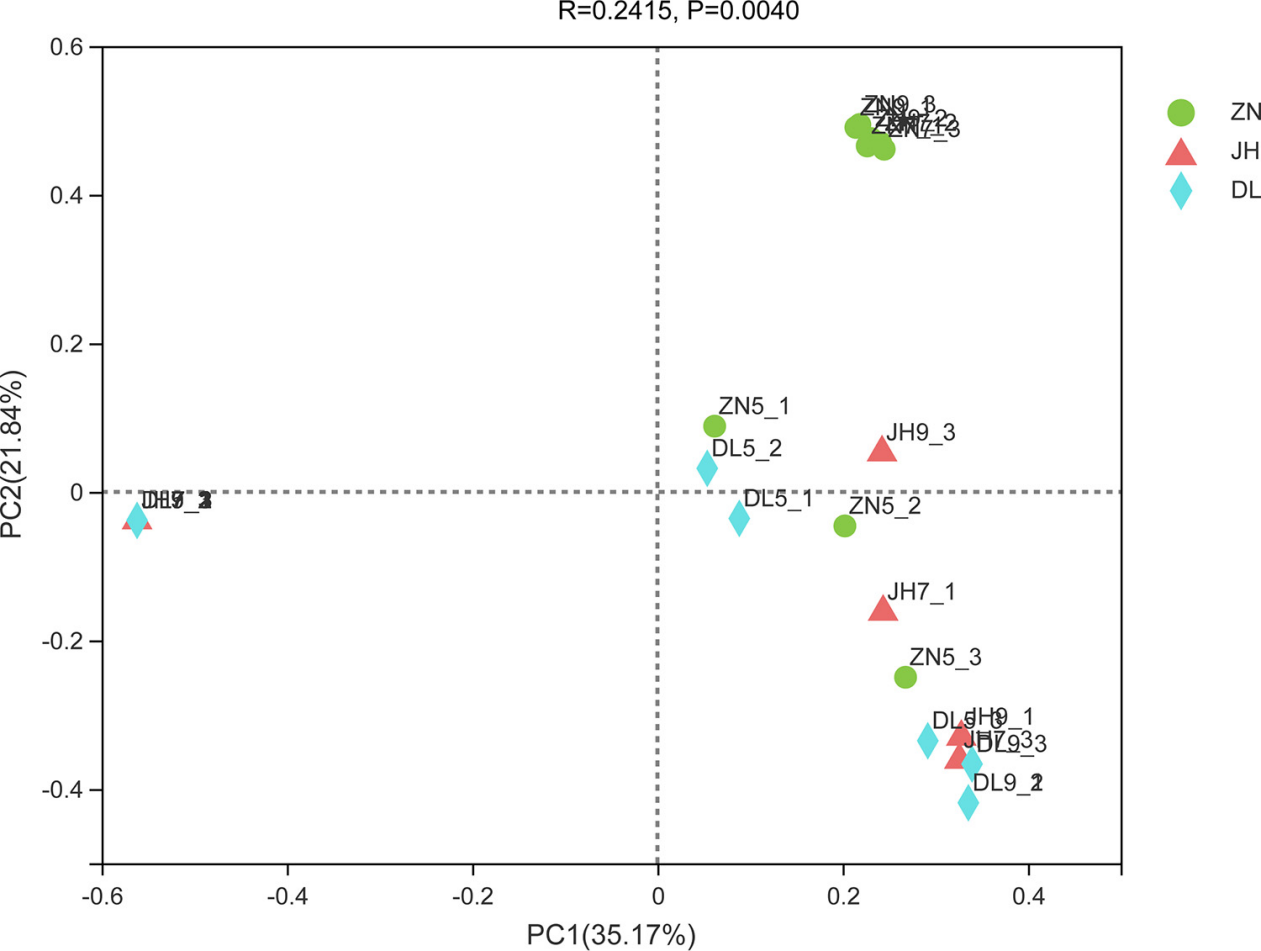
Principal coordinate analysis (PCoA) of AMF communities in different soil samples; PCoA plots are based on Bray-Curtis distances at the OTU level (97% sequence similarity).

PCoA demonstrates sample separation at the OTU level sampled from different origins and in different months (Fig. 5). The two principal axes show the trend of variation in the total AMF community and explain more than half of the variation (Bray-Curtis: PCoA1 = 35.17%, PCoA2 = 21.84%).

Samples from three regions in different months clustered together, except for the July and September samples of the ZN region. These showed significant differences, which is consistent with the results of the community bar plot analysis. Also, DL7-3 and JH9-2 clustered together and were significantly different from the other samples.

Permutational multivariate analysis of variance (PERMANOVA) was performed to examine the relationship and significance analysis among variation in AMF communities, environmental factors, and fruit ingredients for different samples. The results showed significant correlations (*P* < 0.01) between the sample source and pH, electrical conductivity (EC), soil organic matter (SOM), TN, TS, FLA, monthly average atmospheric humidity (MAAH), TP, and BET; and some correlations (*P* < 0.05) were also found with SUR, amylase (AMY), AK, LBP, monthly average soil temperature (MAST), monthly minimum temperature (MMinT), AP, URE, monthly average temperature (MAT), protease (PRO), monthly maximum temperature (MMT), and TK.

**FIG 6:**
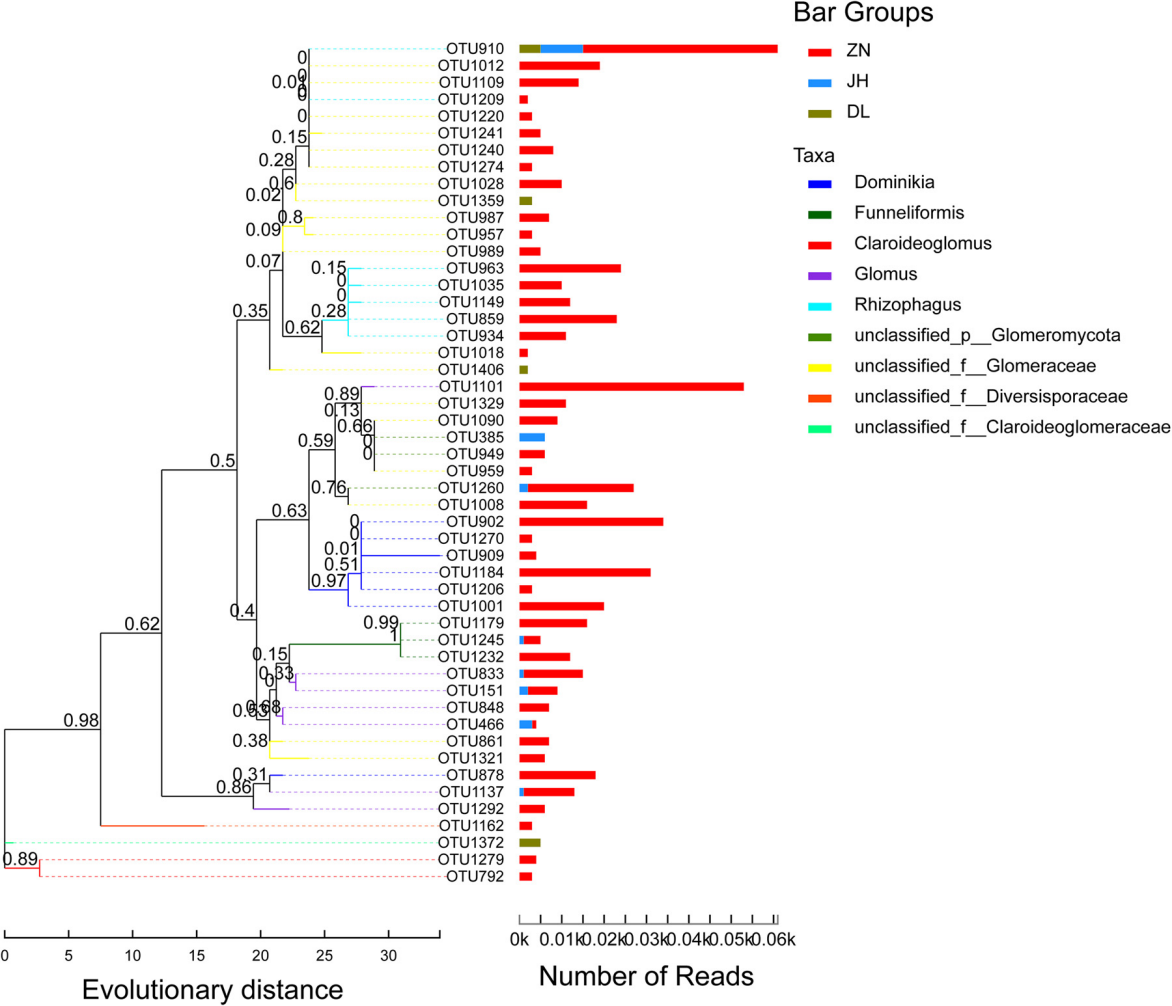
Phylogenetic tree of the OTUs from three different habitats. The tree was constructed at the genus level by selecting the top 50 most abundant OTUs using the maximum parsimony (MP) method.

The phylogenetic positions of the top 50 most abundant OTUs in samples from three different regions were determined according to the 97% similarity cutoff (Fig. 6). We found that these OTUs were evenly distributed in the phylogenetic tree, and *Rhizophagus* sequences were present in all three regions; some sequences also belonged to the unclassified family Glomeraceae. Also, *Glomus* and *Rhizophagus* sequences were higher in the ZN region sample.

### Correlation among AMF communities, *L. barbarum* fruit ingredients, and environmental factors.

For correlation analysis between AMF communities and environmental factors, the JH5-1, JH5-3, JH7-2, JH9-2, DL7-1, DL7-3, and DL7-3 samples that contained fewer AMF species were excluded.

**FIG 7:**
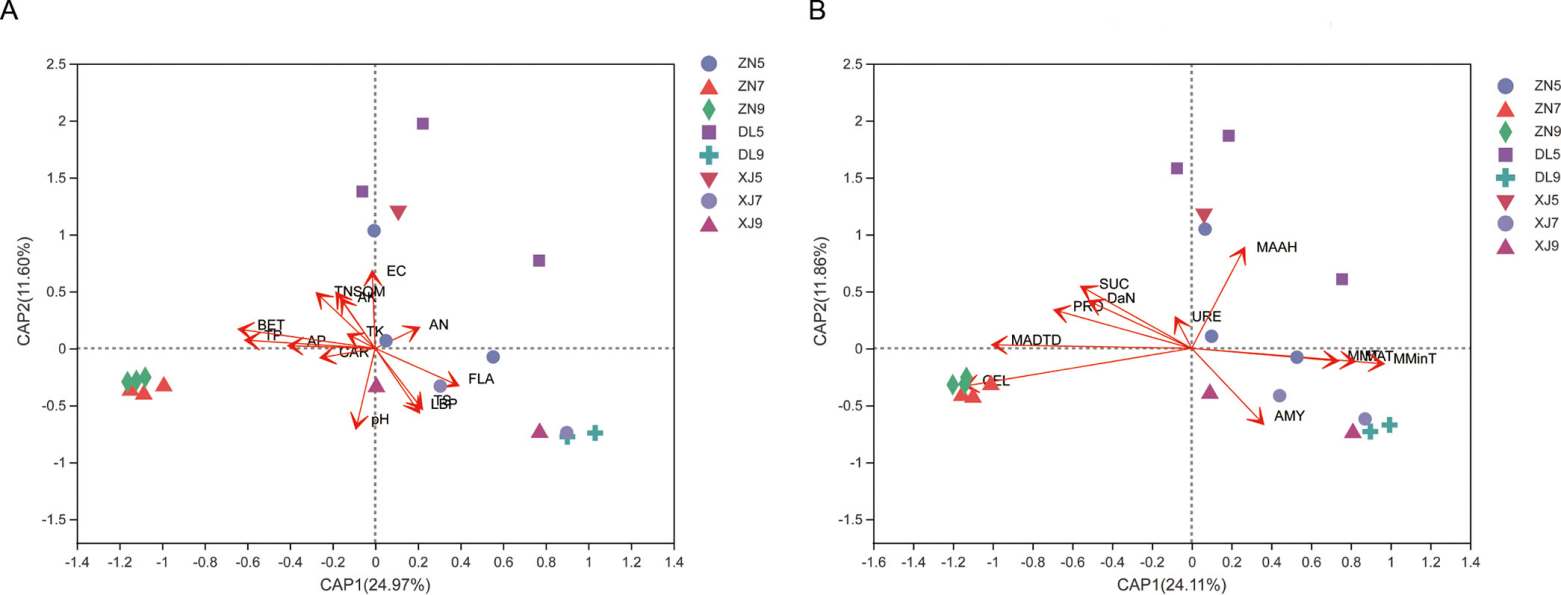
db-RDA correlation analysis among AMF community, environmental factors, and ingredients of *L. barbarum* fruit. (A) Correlations between AMF communities, soil physicochemical properties, and enzymatic activity. (B) The temperature difference and fruit composition content were analyzed at the genus level using the Bray-Curtis distance algorithm. The change in fruit composition of *L. barbarum* was considered a joint influence of AMF communities at different times of the year.

A db-RDA analysis was performed for correlation analysis among AMF communities, environmental factors, and fruits ingredients in samples from different regions and months; 36%, 57%, and 35.97% of the variations in AMF communities are explained by each of the two ranking axes (Fig. 7A and B). Similar trends were found for correlations between environmental factors and fruit in different regions but from the same month; the ZN samples in July and September showed a different correlation with environmental factors and fruit ingredients than the May ZN sample. AMF communities were highly correlated with pH, EC, BRT, and CEL (*P* < 0.01) and significantly correlated with TN, TP, TS, LBP, monthly average daily difference in temperature (MADTD), and MAST (*P* < 0.05; see Table S5 in the supplemental material).

**FIG 8:**
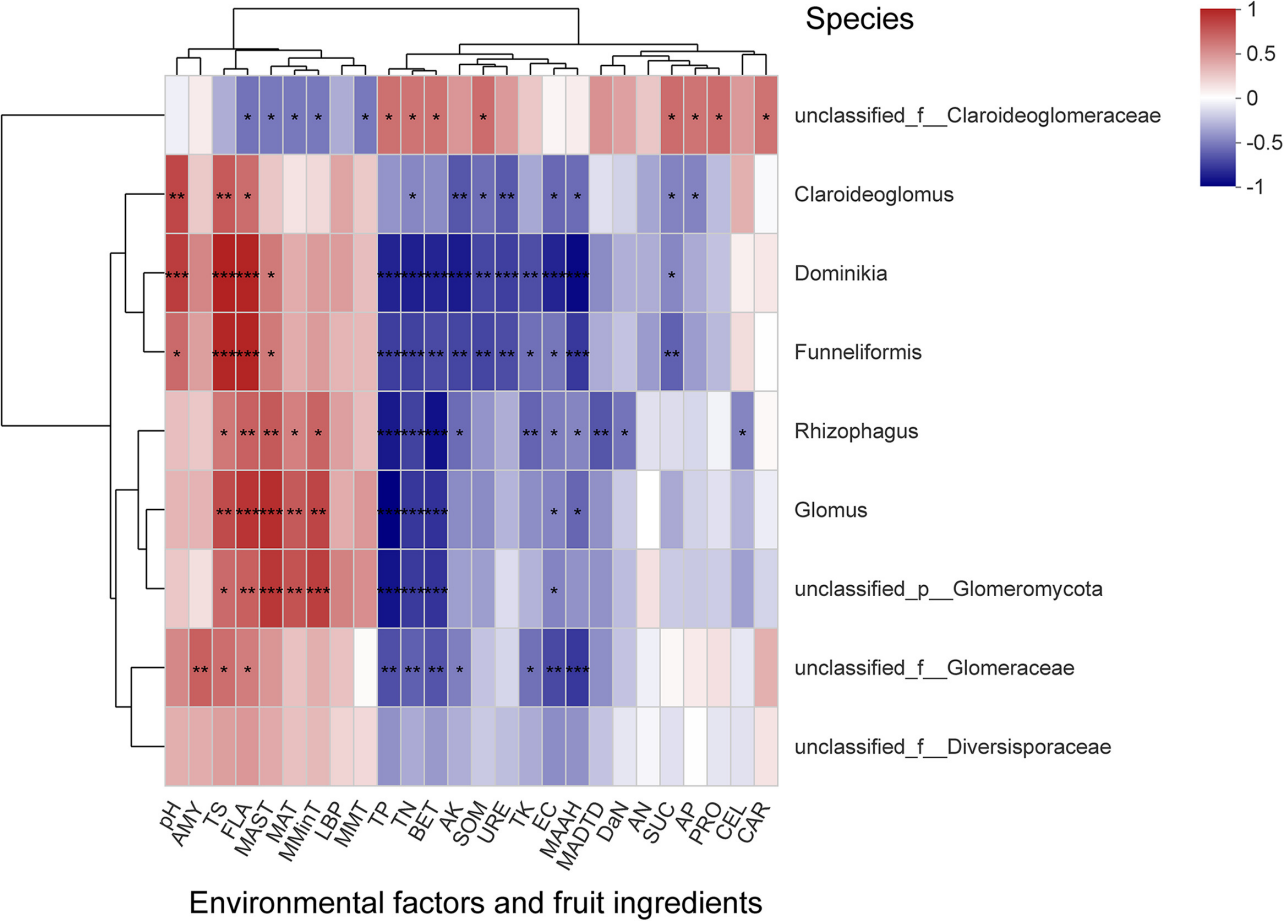
The heatmap shows the Spearman correlation of AMF communities with environmental factors at the genus level; the x and *y* axes correspond to environmental factors and genera, respectively. Different colors indicate R values corresponding to the correlations between individual variables. *, *P* < 0.05; **, *P* < 0.01; ***, *P* < 0.001.

The correlations among environmental factors, fruit ingredients, and AMF communities are reflected at the genus level (Fig. 8), with the exception of two unclassified families (unclassified_f__Claroideoglomeraceae and unclassified_f__Diversisporaceae). The correlations of AMF genera with environmental factors and fruit ingredients were significantly positive for TS, FLA, and significantly negative for TN and EC (see Table S6 in the supplemental material).

Three genera, *Claroideoglomus*, *Dominikia*, and *Funneliformis*, were significantly positively correlated with pH. Two genera, *Dominikia* and *Funneliformis*, were significantly positively correlated with TP, BET, AK, SOM, URE, TK, MAAH, and TN. *Rhizophagus*, *Glomus*, and unclassified_p__Glomeromycota were significantly positively correlated with MAST, MAT, and MMinT, and negatively correlated with TP and BET.

## DISCUSSION

AMF symbiosis provides a range of benefits to the host plant in exchange for plant-produced carbohydrates such as improved nutrition, resistance to soilborne pests and diseases, drought tolerance, heavy metals tolerance, and soil structure (43–45). However, the diversity and richness of AMF communities can vary with geographical regions. A comprehensive understanding of the biotic and abiotic factors affecting AMF communities can provide important insights for sustained agroecosystems (26, 35).

There is no consensus barcoding region for the determination of AMF taxa (46). Internal transcribed spacer (ITS) sequences serve as a theoretical basis for microbial species identification and community analysis as these do not code for ribosome components and are highly variable (41). Therefore, ITS is suggested to be used as the primary fungal barcode marker (47). Previously, a total of 33 AMF species from 11 genera were identified based on morphology in Ningxia province, where the Zhongning area is located (32). Here, we identified a total of 46 OTUs in ZN samples collected in 3 different months. However, only a few AMF OTUs were detected in other regions. Notably, several OTUs belonging to AMF could not be accurately classified, and those could be new AMF species that were absent in the 18S rRNA database Unite (https://unite.ut.ee).

The present study can be considered as a regional survey of typical *L. barbarum* planting areas, with three sampling sites located in northwestern China, at an altitude of 1,123 m, 290 m, and 2,783 m in the Zhongning, Jinghe, and Dulan regions, respectively. More OTUs were detected in the ZN samples showing higher richness and diversity. In Brazil, variation in AMF communities was found along an altitudinal gradient of 700 m (800 m to 1,400m); AMF density and richness were higher at intermediate altitudes, and species composition showed statistical differences at different altitudes (48). This suggests that within a certain range, the highest altitude of sampling sites was only 706 m and elevation had no negative effect on AMF species richness (26). This is consistent with our findings. However, diverse altitudes may have different geographical variations such as temperature, oxygen concentration, sunlight intensity, etc., which might affect AMF community structure.

The *Rhizophagus* genus was present in all regions (ZN 23%, JH 37%, and DL 33%), while ZN soil samples were highly enriched in *Rhizophagus* (23%), *Dominikia* (21%), and *Glomus* (18%). In general, *Rhizophagus* and *Glomus* are abundant in rhizosphere soil samples in both farming and restoration areas (49–53), which is consistent with our results.

In addition, PCoA based on OTU levels showed that samples from different regions clustered together, which is consistent with the results of the species composition analysis. The AMF community in the Zhongning region differed from the other samples in July and September, while months variation had no specific effect on mycorrhizal community structure or diversity (54). Notably, the natural or temporal variability in the AMF community is much higher in the field than that caused by genetically modified (GM) crops (55) because of intense fungal competition for nutrients (56). However, the environmental factors that vary with months (seasons) are complex and diverse; PCoA showed that AMF communities were mainly positively correlated with MMinT, MAT, MAST, and negatively correlated with MAAH. It is evident that temperature and humidity also affected AMF communities; therefore, climate change can significantly alter the community dynamics and ecosystem of AMF (57, 58). Also, the growth of host plants can impact the AMF ecosystem. For instance, AMF symbiosis can ameliorate the impact of saline soil on peanut production (59). Likewise, plant flavonoids can help host plants scavenge free radicals to enhance drought tolerance. However, we found that fruit flavonoid content showed a negative correlation with most AMF species, presumably because AMF can increase plant stress tolerance lowering flavonoid content.

The impact of soil quality on AMF communities is essential for our understanding of ecosystem functioning. Notably, enzyme activity is one of the best indicators of soil nutrients quality and microbial metabolism. In this study, db-RDA analysis revealed significant differences (*P* < 0.01) between the soil samples from three regions; cellulase showed a significant positive correlation with AMF communities. Cellulase degrades cellulose into glucose for microbial use. This suggests that cellulase provides a conducive environment for the growth of AMF communities. Urease content indicates the nitrogen status of the soil. Spearman correlation analysis revealed that three genera, *Claroideoglomus*, *Dominikia*, and *Funneliformis*, were significantly negatively correlated with urease, showing a negative effect of urease and total nitrogen. AMF inoculation is known to increase the soil urease, convertase, and cellulase activities (23). Ammonia, an enzymatic product of urease, is not irreplaceable. However, some studies showed that AMF can acquire nitrogen from both inorganic and organic nitrogen sources and transfer it host plant (60, 61). Nevertheless, AMF, due to its poor exo-enzymatic repertoire, is unlikely to degrade organic compounds on its own (62), and therefore total plant nitrogen and biomass do not generally increase (61).

Mostly arid, barren, and saline soil (with alkalinity) of Northwest China has the highest pH, compared with the other two regions. Especially, the soil of the Zhongning region is poor with lower soil physicochemical indicators, but it has a higher abundance of AMF species. Many studies suggest that soil pH significantly affects the AMF communities (33, 35, 63–66). For example, alkaline soils in forest ecosystems positively affect the AMF communities (67), while acidic soils negatively affect the AMF spore density and evenness (67). This often produces different outcomes. Besides, phosphorus can also greatly impact the AMF community structure (50, 68, 69); however, we did not find the same in this study. Even under low phosphorus conditions in the Zhongning region, effective phosphorus concentration increased significantly after a few months, especially in the high-demand month of July. Perhaps, under low phosphorus availability, AMF colonization stimulated plant roots to release additional carboxylates and alkaline phosphatase (70). Overall, understanding the effects of various environmental factors on AMF communities can provide better insights into host plant growth and better crop yields.

### Conclusions.

We analyzed AMF communities in rhizosphere soil from three high-quality *L. barbarum* producing regions in northwest China and found the highest AMF abundance in the Zhongning region despite poor soils. The results show that rhizosphere soil physiochemical properties, enzyme activity, climatic factors (temperature difference, etc.), and fruit ingredients correlate with AMF communities. Furthermore, different AMF genera (*Claroideoglomus*, *Dominikia*, and *Funneliformis*) correlated to environmental factors and fruits ingredients in a similar manner affecting the TS and FLA contents in the fruits of *L. barbarum*.

## MATERIALS AND METHODS

### Sampling design and collection.

The pure seed and healthy tree of “Ning Qi 1” Chinese *L. barbarum* single plant were raised using shoot cuttings breeding technology in Zhongning (Ningxia Province), Jinghe (Xinjiang Province), and Dulan (Qinghai Province) counties (Table 2). TT-QXZ weather stations were installed at the three test sites to collect and record weather and temperature data in a continuous and real-time manner. Before the main experiments were conducted, young fibrous roots of *L. barbarum*, collected from the three test regions, were stained using the Trillium blue method to observe the presence of arbuscules and intraradical hyphae under a light microscope (Olympus CX21). This confirmed that rhizosphere AMF from all three regions could infest *L. barbarum* and produce symbiotic structures.

**TABLE 2:** Details of sample collection^*a*^

Studied sites	Province	Location	Soil type	Acquisition subjects	Sample codes	Latitude (N)	Longitude (E)	Altitude	Depth
1	Ningxia	Zhongning County	Normal arid soil	*L. Barbarum*(NingQi 1)	ZN5 ZN7 ZN9	37°16′26″	105°27′16″	1123m	20 to 40 cm
2	Xinjiang	Jinghe County	Normal saline soil	*L. Barbarum*(NingQi 1)	JH5 JH7 JH9	44°20′28″	82°32′7″	290m	20 to 40 cm
3	Qinghai	Dulan County	Cold arid soil	*L. Barbarum*(NingQi 1)	DL5 DL7 DL9	36°13′37″	92°15′19″	2783m	20 to 40 cm

a Sample codes 5, 7, and 9 represent the sample collection months May, July, and September, respectively. Each sample contained three replicates that were numbered separately. Soil type was obtained from https://www.osgeo.cn.

Three sites were randomly selected for the experimental survey, and the soil samples were collected at three different time points in May (berry budding stage), July (berry blooming stage), and September (berry fruiting stage). A clean shovel was used to remove surface foliage, stones, and debris to collect soil samples at 20 cm to 40 cm depth, which were well marked and stored in self-sealing bags. Three random soil samples were collected each month at one sampling site and then divided into two parts. Those were placed into two separate sterilized 50-mL centrifuge tubes in an ultra-low temperature (−80°C) refrigerator for fungal 18S rDNA high-throughput sequencing and soil physicochemical parameters analysis, respectively. Rhizosphere soil samples were collected from three areas in 3 months with three replicates, making a total of 27 samples. *L. barbarum* produces summer fruits with indefinite inflorescences at full flowering and fruiting stages. The summer fruits were harvested for five to seven crops, and autumn fruits were collected after summer dormancy was over. The crop with the most fruits was selected to represent the year’s yield and the analysis of fruit's active ingredients.

### DNA extraction and PCR amplification.

Soil DNA was extracted from all 27 samples using 0.5-g soil samples and FastDNA Spin Kit for Soil following the manufacturer’s instructions (MP Biomedicals, USA). DNA purity, concentration, and integrity were determined by NanoDrop 2000 UV-vis spectrophotometer (Thermo Scientific, Wilmington, USA), and 1% agarose gel electrophoresis, respectively. The PCR was performed to amplify 18S rDNA fungal microbial fragments with ITS1F (5′-CTTGGTCATTTAGAGGAAGTAA-3′) and ITS2R (5′-GCTGCGTTCTTCATCGATGC-3′) primers corresponding to the region ITS1 (length 350 bp). PCR was carried out in a 20-μL reaction mixture consisting of a 10 ng DNA template, 0.4 μL FastPfu Polymerase (TransGen, China), 0.8 μL (5 μM) Forward Primer, 0.8 μL (5 μM) Reverse Primer, 4 μL 5×FastPfu Buffer, 2 μL 2.5 mM dNTPs, 0.2 μL BSA, and ddH2O to complete the remaining volume. PCR conditions were as follows: 3 min of denaturation at 95°C; 30 seconds at 95°C for 34 cycles; 30 seconds primer annealing at 55°C; 45 seconds extension at 72°C, 10 min final extension at 72°C and lastly 10°C until removed for storage. Three PCR product replicates were produced for each sample and then mixed. The products were analyzed using 2% agarose gel electrophoresis and then gel purified using the AxyPrep DNA Gel Extraction Kit (Axygen Biosciences, Union City, CA, USA). Finally, the PCR products were quantified by a Quantus Fluorometer (Promega, USA). The samples were diluted based on the amount required for sequencing.

### Illumina MiSeq sequencing.

The purified amplicons were combined in equal amounts, and paired-end sequencing was performed on the Illumina MiSeq PE300 platform (Illumina, San Diego, USA) following the standard procedures of Majorbio Bio-Pharm Technology Co. Ltd. (Shanghai, China; see Table S7 in the supplemental material).

### Processing of sequencing data.

The raw reads of 18S rRNA gene sequencing were demultiplexed and quality filtered with fastp version 0.20.0 (71) and then merged with FLASH version 1.2.7 (72). A window size of 50 bp was set, and any 300 bp read with an average quality score <20 points at any site was truncated. After truncation, reads <50 bp were discarded. Also, the reads containing ambiguous characters were discarded. Only overlapping sequences >10 bp were assembled with the maximum mismatch ratio of 0.2. Reads not following this criterion were discarded. Finally, the samples were distinguished based on barcodes and primers; sequence orientation was adjusted with exact matches for barcodes and a maximum mismatch of two nucleotides in primers. OTUs with a 97% similarity cutoff were clustered using UPARSE version 7.1 (73) to identify and remove chimeric sequences. For the taxonomy analysis of representative sequences, the 18S rRNA database Unite (https://unite.ut.ee) was screened with RDP classifier version 2.2 (74) with a confidence threshold of 0.7.

### Environmental factors (physicochemical properties, enzymatic activities, and temperature) and fruit ingredients.

Nine major soil physicochemical factors affecting microbial communities were analyzed (75, 76). The soil pH was determined by the potentiometric method based on hydrogen ion activity in solution; EC was measured by passing a 1-mm screen with a water-soil mass ratio of 5:1. SOM was determined using the potassium dichromate-sulfuric acid method. TN and rapid AN contents were determined by Kjeldahl and alkaline diffusion methods, respectively. TP and rapid AP contents were estimated by sulfuric acid-perchloric acid decoction method, and sodium bicarbonate methods, respectively. TK and rapid AK contents were determined by sodium hydroxide fusion-flame and ammonium acetate flame photometry methods, respectively. The sodium phenol-sodium hypochlorite colorimetric method was used to determine URE, and the 3,5-dinitrosalicylic acid colorimetric method was used to determine AMY, sucrase (SUC), and CEL. PRO content was determined by Ninhydrin colorimetric method. The five active *L. barbarum* fruit ingredients, TS, LBP, BET, FLA, and carotenoids (CAR) were measured by double-beam UV-Vis spectrophotometry.

All three test sites were installed with TT-QXZ type weather stations with a probe depth of 30 cm to record the temperature. The meteorological data were collected continuously in real-time. A total of nine items, the MAT, MMT, MMinT, MADTD, diurnal temperature difference (DaN), MAAH, and MAST during the growth and development period of *L. barbarum* were used for data analysis. The abbreviations of the indicators are summarized in (Table 3).

**TABLE 3:** Explanation of environmental factors and fruit composition nomenclature abbreviations

No.	Environmental factors			*L. barbarum* fruit ingredients
	Soil physicochemical properties	Soil enzyme activity	Temp and humidity	
1	pH, Hydrogen ion concn index	AMY, amylase(mg/g)	MAT, monthly avg temp(°C)	TS, total sugar(g/100g)
2	EC, electrical conductivity(mS/cm)	CEL, cellulase(mg/g)	MMT, monthly maximum temp(°C)	LBP, *L. barbarum* polysaccharides (g/100g)
3	SOM, Soil organic matter(g/kg)	URE, urease(mg/g)	MMinT, monthly minimum temp(°C)	BET, betaine(g/100g)
4	TN, Total soil nitrogen(g/kg)	PRO, Protease(mg/g)	MADTD, monthly avg daily difference in temp(°C)	FLA, flavonoids (g/100g)
5	TP, Total phosphorus(g/kg)	SUR, sucrase(mg/g)	DaN, diurnal temp difference(°C)	CAR, carotenoids (g/100g)
6	TK, Total potassium(g/kg)		MAAH, monthly avg atmospheric humidity(%)	
7	AN, rapid available nitrogen(mg/kg)		MAST, monthly avg soil temp(°C)	
8	AP, rapid available phosphorus(mg/kg)			
9	AK, rapid available potassium(mg/kg)			

### Statistical analysis.

The clumped mycorrhizae belonging to the phylum Glomeromycota were screened from respective samples. To study the diversity index, each sample was sampled flat for community diversity analysis, and dilution curves were constructed with a minimum number of sample sequences using the R language tool. To analyze the species richness of the samples, we calculated S_obs_ (index for the actual number of OTUs), ACE (index for richness and evenness of species composition), and Chao (index for number of OTUs) indices using mothur (version v.1.30.1) (http://www.mothur.org/wiki/Schloss_SOP#Alpha_diversity) software. Shannon and Simpson indices, which reflect the community and spectral diversity, were also calculated. PD was used to measure the biodiversity of the microbial communities.

The samples from three regions were analyzed for comparative analysis of species composition by selecting OTUs with 97% similarity. Venn diagrams were constructed to compare divergent and shared species, and community histograms were plotted at the genus level based on OTUs classification. The classification was statistically and graphically presented using R language tools. Given that the sample species abundance did not conform to a normal distribution, the Kruskal-Wallis rank-sum test without overall restrictions and the *post hoc* test for multiple test correction were applied. The Tukey-Kramer method (which requires the same number of samples) was used to analyze the variability between groups with a confidence interval of 0.95. To further assess the similarity of community composition between different samples, the Bray-Curtis distance algorithm was used at the OTU level considering the abundance and presence of species. ANOSIM test was used to find the difference between groups, and the number of Monte Carlo random permutations was set to 999 as default for PCoA analysis. PERMANOVA was used to decompose the total variance using the semi-metric Bray-Curtis algorithm to analyze the effect of different grouped environmental factors on sample differences. The statistical significance of the divisions was analyzed using Monte Carlo random permutations (999 times) in statistical analysis.

To understand the history and sequence of biological evolutionary processes in AMF communities of the three regions, evolutionary trees were systematically constructed at the OTU level using maximum parsimony (MP) method in Mega software (version 10.0 https://www.megasoftware.net/).

SPSS 20.0 statistical analysis software was used to test the significance of the differences between the soil physiochemical indicators and the active ingredients of *L. barbarum* fruits in different months using Tukey test or one-way ANOVA (*P* <0.05). To analyze the relationship between AMF communities and environmental factors, db-RDA (distance-based redundancy analysis) analysis was chosen for its ability to address the limitation of data type (29). The Bray-Curtis distance algorithm was chosen for species-genus level analysis and RDA (redundancy analysis) was used to identify the relationships between principal coordinates (species data) and dummy variables (models). Correlation coefficients between the fruit ingredients, environmental factors, and OTUs were calculated at the genus level by R software (pheatmap package) using Spearman rank correlation coefficients, and environmental factors and species hierarchical clustering methods are both average.

### Data availability.

The raw reads have been stored in the NCBI Sequence Read Archive (SRA) database (PRJNA759085). Temporary Submission ID: SUB10211634.
