# Supplementary material for: Illumina MiSeq Sequencing Reveals Correlations among Fruit Ingredients, Environmental Factors, and AMF Communities in Three Lycium Barbarum Producing Regions of China
Source: Microbiol Spectr. 2022 Mar 2;10(2):e02293-21. doi: 10.1128/spectrum.02293-21 (PMC8941938; doi:10.1128/spectrum.02293-21)
Supplement: SUPPLEMENTAL FILE 1 — Supplemental material. Download SPECTRUM02293-21_Supp_1_seq2.pdf, PDF file, 0.3 MB [file spectrum02293-21_supp_1_seq2.pdf]

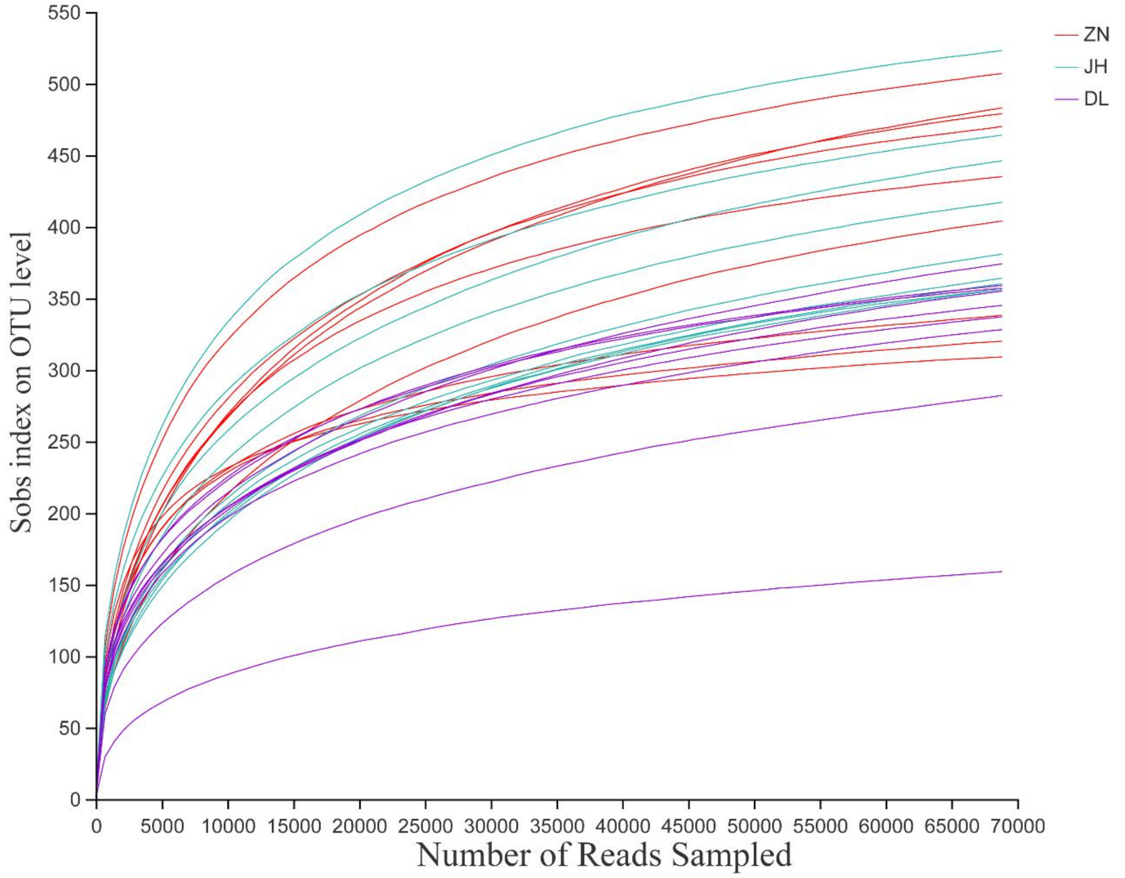

Figure S1. Dilution curves of rhizosphere soil samples from three regions, Red represents Zhongning(ZN)County, purple represents Jinghe(JH) County and green represents Dulan(DL) County, samples from all three areas contain rhizosphere (20-40 cm) soil from three periods (May, July and September)

Table S1. Chemical properties of the sampled soils from the three regions.

	PH	EC(mS/cm)	SOM(g/kg)	TN(g/kg)	TP(g/kg)	TK(g/kg)	AN(mg/kg)	AP(mg/kg)	AK(mg/kg)
ZN5	8.60±0.02Aa	0.06±0.00Ba	2.81±0.04Cb	0.30±0.00Ca	0.93±0.00Ca	18.86±0.06Ba	18.00±1.00Cb	11.66±0.21Cc	80.00±0.00Cb
ZN7	8.43±0.01Ac	0.04±0.00Cb	3.82±0.18Ca	0.25±0.00Cab	0.47±0.00Cb	18.06±0.06Cb	126.66±2.33Ba	39.43±1.01Ba	98.33±1.66Ba
ZN9	8.52±0.00Ab	0.06±0.00Ca	3.67±0.08Ca	0.28±0.00Cb	0.45±0.00Cc	15.93±0.06Cc	10.00±0.57Cc	23.00±0.11Bb	75.00±0.00Cc
JH5	8.18±0.00Ba	0.13±0.00Ac	12.93±0.08Ba	0.80±0.00Ba	1.36±0.01Ba	23.30±0.10Aa	96.66±1.45Ab	32.00±0.30Ba	151.66±1.66Bb
JH7	7.52±0.02Cc	0.38±0.00Aa	11.80±0.20Bb	0.81±0.01Ba	0.79±0.00Bb	21.93±0.06Ab	122.66±3.17Ba	26.13±0.17Cb	206.66±1.66Aa
JH9	8.01±0.00Bb	0.14±0.00Bb	11.90±0.05Bb	0.73±0.00Bb	0.81±0.00Bb	21.66±0.16Ab	55.33±0.33Bc	16.83±0.14Cc	143.33±1.66Ac
DL5	8.16±0.00Ba	0.12±0.00Ab	16.70±0.11Ab	1.04±0.00Ab	1.87±0.01Aa	19.13±0.06Bb	89.33±2.33Bb	163.66±3.38Ab	168.33±1.66Ab
DL7	7.82±0.00Bc	0.09±0.00Bc	18.76±0.03Aa	1.31±0.02Aa	1.18±0.01Ab	20.06±0.13Ba	221.33±1.20Aa	176.00±2.64Aa	203.33±1.66Aa
DL9	8.00±0.00Bb	0.16±0.00Aa	14.46±0.13Ac	0.87±0.00Ac	1.05±0.00Ac	17.33±0.16Bc	66.33±0.88Ac	70.13±2.21Ac	125.00±2.88Bc

Different upper and lowercase letters indicate a significant difference among different regions in the identical months and different months in the same region, respectively. All data are mean ± standard error according to the Tukey test($P<0.05$). T, total; A, available.

Table S2. Soil enzyme activity in three areas.

	AMY(mg/g)	CEL(mg/g)	URE(mg/g)	PRO(mg/g)	SUR(mg/g)
ZN5	2.99±0.00Cc	16.07±0.00Aa	0.17±0.00Cc	0.22±0.00Bb	7.23±0.00Cc
ZN7	3.07±0.00Bb	0.09±0.00Cc	0.30±0.00Ca	0.22±0.00Bb	21.14±0.00Ba
ZN9	10.17±0.00Aa	0.68±0.00Ab	0.25±0.00Cb	0.33±0.00Ba	20.41±0.00Cb
JH5	3.50±0.00Ba	1.06±0.01Ca	0.55±0.00Bb	0.10±0.00Cc	29.07±0.00Bb
JH7	0.00±0.00Cb	0.45±0.00Bc	0.51±0.00Bc	0.24±0.00Ab	17.46±0.00Cc
JH9	0.00±0.00Cb	0.62±0.00Ab	0.57±0.00Aa	0.27±0.00Ca	30.78±0.00Ba
DL5	5.22±0.00Aa	1.13±0.00Bb	0.57±0.01Aa	0.55±0.00Aa	131.96±0.00Ab
DL7	4.78±0.00Ab	3.11±0.00Aa	0.55±0.00Ab	0.10±0.00Cc	248.35±0.00Aa
DL9	3.95±0.00Bc	0.16±0.12Bc	0.33±0.00Bc	0.42±0.00Ab	107.90±0.00Ac

Different upper and lowercase letters indicate significant differences among different regions in the identical month and different months in the same region, respectively. All data are mean ± standard error according to Tukey's test ($P<0.05$).

Table S3. Relevant temperature indicators in the three regions

	MAT(℃)	MMT(℃)	MMinT(℃)	MADTD(℃)	DaN(℃)	MAAH(%)	MAST(℃)
ZN5	17.60±1.42Ab	33.91±1.44Aab	2.79±0.39Bb	13.57±1.37Aa	4.59±0.05Ba	30.31±1.10Ba	18.49±1.16Ab
ZN7	25.16±0.63Aa	38.83±1.34Aa	13.73±0.95Aa	12.19±1.36Aa	4.39±0.49Bab	37.51±5.65Aa	25.60±0.50Aa
ZN9	17.87±1.28Ab	30.50±1.17ABb	4.80±1.68Ab	12.03±1.74Aa	3.04±0.37Ab	32.54±6.16Ba	20.74±0.70Ab
JH5	18.81±0.36Ab	34.47±0.98Ab	5.30±0.58Ab	11.77±1.30Aa	8.23±0.29Aa	40.29±0.38Ab	18.64±0.20Ab
JH7	27.27±0.48Aa	40.47±1.34Aa	15.50±0.82Aa	14.26±1.00Aa	9.91±0.44Aa	45.59±0.87Aab	26.95±0.68Aa
JH9	17.73±0.55Ab	33.00±1.04Ab	3.80±2.25Ab	12.66±1.79Aa	6.48±2.74Aa	53.74±4.16Aa	19.91±0.62Ab
DL5	11.52±0.83Bb	25.67±2.14Ba	-0.87±0.35Cb	14.91±0.42Aa	7.74±0.10Aa	31.13±1.86Bb	11.38±0.03Bc
DL7	17.74±0.69Ba	31.17±0.44Ba	7.13±1.50Ba	13.30±0.71Aa	5.85±0.52Bb	48.50±0.30Aa	18.67±1.00Ba
DL9	11.76±1.28Bb	26.90±1.25Ca	-0.57±1.28Ab	12.77±1.18Aa	4.27±0.17Ac	45.00±3.77ABa	14.51±0.42Bb

Different upper and lowercase letters indicate significant differences among different regions in the identical month and different months in the same region, respectively. All data are mean ± standard error according to Tukey's test (P<0.05). Numbers 1-3 denote the months of May, July, and September, respectively.

Table S4. Comparison of the fruit ingredients of *L.barbarum* in the three regions.

	TS /(g/100g)	LBP /(g/100g)	BET /(g/100g)	FLA /(g/100g)	CAR /(g/100g)
ZN	48.58±0.29A	2.70±0.01A	0.82±0.02A	0.15±0.01A	0.37±0.01B
JH	47.36±0.09B	2.72±0.10A	0.86±0.03A	0.13±0.01AB	0.24±0.00C
DL	46.18±0.18C	1.84±0.10B	0.94±0.03A	0.11±0.00B	0.42±0.00A

Different uppercase letters indicate the significant differences in the fruit composition of *L. barbarum* in the three regions. All data are mean ± standard error according to Tukey's test(P<0.05).

Table S5. PERMANOVA examines the relevance of environmental factors and fruit ingredients for sample differences (AMF communities) in different subgroups

Characteristics	R^2	P-value	Padjust
pH	0.173	0.001	0.004
EC	0.145	0.001	0.004
SOM	0.219	0.001	0.004
TN	0.234	0.001	0.004
TS	0.225	0.001	0.004
FLA	0.204	0.001	0.004
MAAH	0.162	0.001	0.004
TP	0.203	0.002	0.006
BET	0.195	0.002	0.006
SUR	0.155	0.005	0.013
AMY	0.146	0.009	0.020
AK	0.156	0.010	0.020
LBP	0.151	0.010	0.020
MAST	0.156	0.012	0.022
MMinT	0.133	0.014	0.024
AP	0.131	0.017	0.028
URE	0.128	0.023	0.033
MAT	0.120	0.023	0.033
PRO	0.117	0.034	0.046
MMT	0.118	0.035	0.046
TK	0.116	0.039	0.048
CEL	0.092	0.063	0.074
CAR	0.077	0.137	0.155
DaN	0.074	0.172	0.186
MADTD	0.067	0.227	0.236
AN	0.037	0.686	0.686

Table S6. AMF community, environmental factors, and db-RDA analysis of *L. barbarum* fruit ingredients.

Characteristics	R^2	P-value
pH	0.515	0.002
EC	0.463	0.004
SOM	0.264	0.074
TN	0.306	0.049
TP	0.394	0.017
TK	0.025	0.774
AN	0.073	0.497
AP	0.169	0.199
AK	0.208	0.125
TS	0.327	0.042
LBP	0.357	0.028
BET	0.457	0.008
FLA	0.257	0.091
CAR	0.071	0.543
AMY	0.169	0.223
CEL	0.439	0.003
URE	0.020	0.838
PRO	0.173	0.199
SUR	0.178	0.181
MAT	0.205	0.155
MMT	0.164	0.228
MMinT	0.286	0.059
MADTD	0.303	0.047
DaN	0.128	0.320
MAAH	0.261	0.075
MAST	0.325	0.039

Table S7. Sample information statistics

Sample	Seq_num	Base_num	Mean_length	Min_length	Max_length
JH5_1	74846	17920203	239.427665	143	422
JH5_2	73824	17810215	241.252371	144	352
JH5_3	71824	17405677	242.337895	149	419
JH7_1	69453	15956144	229.74017	143	397
JH7_2	73078	16642299	227.733367	143	389
JH7_3	70611	16443964	232.881053	142	382
JH9_1	74676	17118830	229.241389	142	424
JH9_2	69932	16072956	229.836927	157	431
JH9_3	73423	17038088	232.053825	148	430
NX5_1	74994	17504888	233.41718	160	412
NX5_2	74800	17154792	229.342139	168	412
NX5_3	72811	16797697	230.702737	142	424
NX7_1	71679	16501782	230.217804	146	424
NX7_2	71054	16334078	229.882596	156	413
NX7_3	72734	16841209	231.545206	149	424
NX9_1	74753	17225865	230.437106	142	412
NX9_2	74503	17637196	236.731353	141	368
NX9_3	71696	16674246	232.568707	157	421
QH5_1	70940	17369176	244.843191	141	428
QH5_2	70225	17411663	247.941089	141	422
QH5_3	71666	17153476	239.353054	141	422
QH7_1	72437	16855514	232.69205	141	412
QH7_2	74741	17070517	228.39562	142	412
QH7_3	70310	16826263	239.315361	144	412
QH9_1	71794	17270686	240.558905	141	427
QH9_2	72002	17022252	236.4136	140	432
QH9_3	74491	18776389	252.062518	141	424
